# Supplementary material for: Splice-Junction-Based Mapping of Alternative Isoforms in the Human Proteome
Source: Cell Rep. Author manuscript; Available in PMC 2020 Jan 15. (PMC6961840; doi:10.1016/j.celrep.2019.11.026)
Supplement: 2 [file NIHMS1546469-supplement-2.zip › DF1/PXD010154/Heart/Heart_53_MYBPC3_TDSHEDTGILDF.pdf]

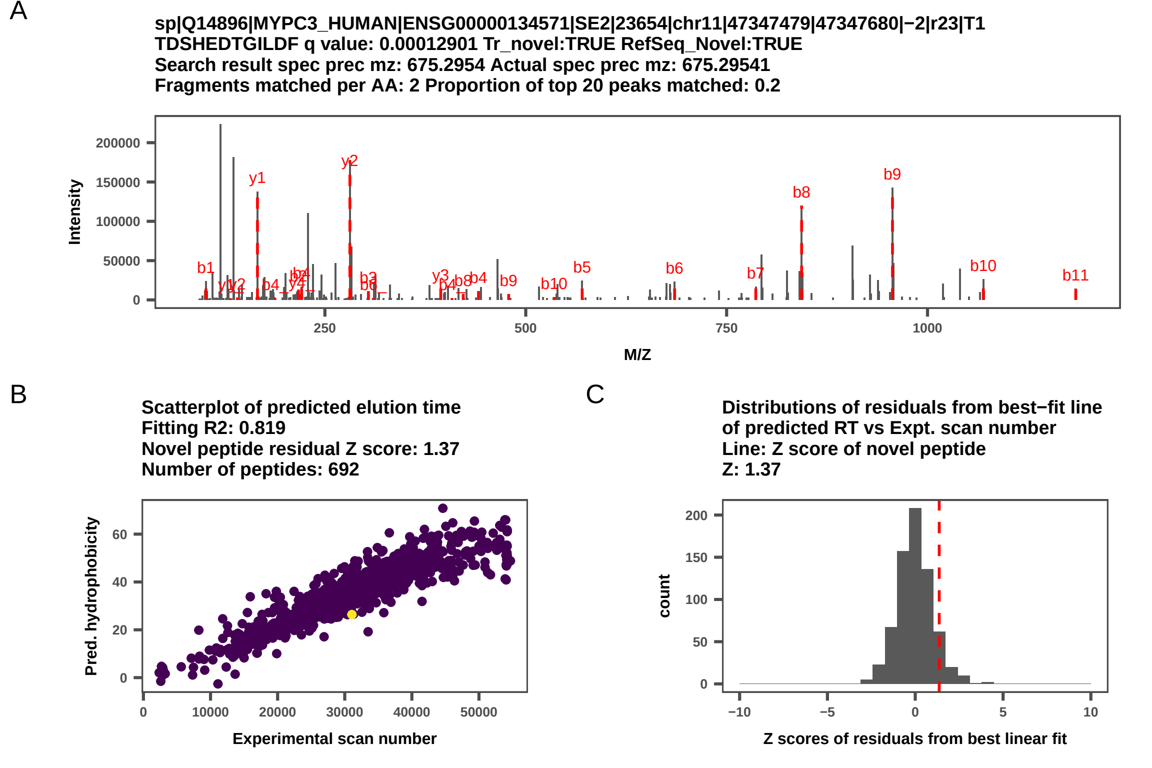
A
sp|Q14896|MYPC3_HUMAN|ENSG00000134571|SE2|23654|chr11|47347479|47347680|−2|r23|T1
TDSHEDTGILDF q value: 0.00012901 Tr_novel:TRUE RefSeq_Novel:TRUE
Search result spec prec mz: 675.2954 Actual spec prec mz: 675.29541
Fragments matched per AA: 2 Proportion of top 20 peaks matched: 0.2
Intensity
200000
150000
100000
50000
0
y1
y2
b1
b5
b6
b7
b8
b9
b10
b11
b3
y3
250
500
750
1000
M/Z
B
Scatterplot of predicted elution time
Fitting R2: 0.819
Novel peptide residual Z score: 1.37
Number of peptides: 692
Pred. hydrophobicity
60
40
20
0
10000
20000
30000
40000
50000
Experimental scan number
C
Distributions of residuals from best−fit line
of predicted RT vs Expt. scan number
Line: Z score of novel peptide
Z: 1.37
count
200
150
100
50
0
−10
−5
0
5
10
Z scores of residuals from best linear fit
